# Association Between the Percentage of US Drug Sales Subject to Inflation Penalties and the Extent of Drug Price Increases

**DOI:** 10.1001/jamanetworkopen.2020.16388

**Published:** 2020-09-11

**Authors:** Sean Dickson

**Affiliations:** 1 West Health Policy Center, Washington, DC

## Abstract

**Importance:**

Policy makers have proposed levying penalties for drug price increases that are higher than the rate of inflation for drugs covered in the US Medicare program, but research is lacking regarding the ways in which manufacturers might respond to those penalties. An understanding of manufacturers’ responses to existing inflation penalties could inform such policy discussions.

**Objective:**

To estimate the association of existing inflation penalties in the US 340B Drug Pricing Program with manufacturer pricing behavior in the Medicare Part D program and associated changes in Medicare pharmacy expenditures.

**Design, Setting, and Participants:**

This study included a pooled cross-sectional time-series analysis of price changes for Medicare Part D drugs used annually by more than 5000 beneficiaries between January 1, 2013, and December 31, 2017. The percentage of Medicare Part D sales subject to inflation penalties in the 340B program was used to perform a regression analysis to estimate the association of inflation penalties with annual drug price changes. The 340B program requires manufacturers to sell their drugs at a lower price to safety-net health care organizations; this lower price includes a base discount and an additional discount equal to the amount of any price increase that is higher than the rate of inflation. Sales to these 340B-eligible health care organizations represent the market share of a drug subject to inflation penalties. Health care organization–level claims data were obtained from the Medicare Provider Utilization and Payment Data–Part D Prescriber database, and organizations were matched to the Office of Pharmacy Affairs Information System of the Health Resources and Services Administration to identify those organizations that were eligible for the 340B program. Price change and drug use data were obtained from the Medicare Part D Drug Spending Dashboard. Name-brand drugs were included in the analysis if they did not have generic competition and were used by more than 5000 individuals with Medicare Part D in 1 calendar year. Data analysis was conducted from January 1 to February 28, 2020.

**Main Outcomes and Measure:**

Annual price change was the primary outcome measure; spending changes associated with lower price increases were also estimated. Sales percentage subject to inflation penalties was the primary independent variable, and formulary classification was a control variable.

**Results:**

Of 2148 brand-name drugs included in the database, 606 drugs were used by more than 5000 beneficiaries annually, with a mean sales percentage subject to inflation penalties of 12.1%. A higher sales percentage subject to inflation penalties was associated with lower annual price increases (between-effects coefficient, −0.114; 95% CI, −0.205 to −0.023; *P* = .01; fixed-effects coefficient, −0.380; 95% CI, −0.466 to −0.294; *P* < .001). Lower price increases owing to inflation penalties were estimated to be associated with a reduction in Medicare Part D pharmacy expenditures of $7.1 billion between 2013 and 2017.

**Conclusions and Relevance:**

In this cross-sectional study, increases in the percentage of drug sales subject to inflation penalties were associated with lower annual price increases. Broader application of inflation penalties may help to reduce drug price increases and decrease overall drug spending.

## Key Points

**Question:**

Are penalties for drug price increases higher than the rate of inflation associated with the extent of drug price increases in the US?

**Findings:**

In this cross-sectional study of 606 brand-name drugs that were used annually by more than 5000 individuals with Medicare Part D from 2013 to 2017, increases in the percentage of drug sales subject to inflation penalties were associated with lower drug price increases. These lower price increases were associated with a reduction in Medicare Part D pharmacy expenditures of $7 billion over the period.

**Meaning:**

The findings suggest that penalties for drug price increases that are higher than the rate of inflation may constrain drug price increases and drug spending.

## Teaser

This cross-sectional study uses data from the Medicare Provider Utilization and Payment Data files to examine the association of existing inflation penalties with manufacturer pricing behavior in the Medicare Part D program and changes in Medicare pharmacy expenditures between 2013 and 2017.

## Introduction

Legislation introduced in 2019 aimed to restrict drug price increases by requiring drug manufacturers to pay a rebate to the Medicare program to offset price increases that are greater than the rate of inflation.^1,2^ After the introduction of these pieces of legislation, some commentators asserted that such policies could encourage price increases on drug sales outside of the Medicare program, shifting costs from the Medicare program to the commercial insurance market.^3^ Supporters of the legislation contended that the rebate requirement would reduce price increases for all purchasers because manufacturers would not increase any of their prices to avoid paying the rebate on Medicare sales.^4^ This study considers the ways in which drug manufacturers have responded to existing inflation penalties to inform policy makers’ consideration of new inflation rebates.

Various existing drug pricing regulations impose inflation-based price adjustments on drug sales. Drug purchases in the Federal Supply Schedule program are subject to inflation limits during the contract period.^5^ The Medicaid Drug Rebate Program requires manufacturers to provide an additional rebate for price increases that are higher than the rate of inflation; this additional rebate means that per-unit Medicaid drug costs decrease as drug manufacturers increase prices.^6^ A third program, the 340B Drug Pricing Program, uses rebate calculation methods from the Medicaid Drug Rebate Program to establish purchase prices for eligible health care organizations, which include nonprofit hospitals and clinics that meet certain federal criteria based on patient population characteristics and funding sources.^7^ To qualify for reimbursement of any of its products through the Medicaid Drug Rebate Program or the Medicare Part B programs, a drug manufacturer must sell its products to 340B-eligible health care organizations (referred to as covered entities) at a lower price, which includes a base discount and an additional discount equal to the amount of any price increase that is higher than the rate of inflation (ie, an inflation penalty).^8^ Sales to these 340B-eligible health care organizations represent the market share of a drug subject to inflation penalties.

The proportion of inflation-penalized sales to covered entities compared with total sales within the Medicare Part D program (referred to as the inflation-penalized sales percentage) can be used to evaluate whether drugs with a larger share of sales subject to inflation penalties have different pricing behavior than drugs with a smaller share of sales subject to inflation penalties. Similar to commentators who have expressed concerns about the proposed Medicare inflation rebate, some analysts have suggested that the inflation penalty discount in the 340B program may encourage drug manufacturers to implement higher price increases than they would in the absence of such a discount.^9,10^ Other commentators have asserted that this discount may mitigate manufacturers’ price increases.^11^ In this study, I examined the association between the inflation-penalized sales percentage of drugs and historical price increase behavior to inform the broader policy debate about the use of inflation-based price controls. I also estimated the differences in Medicare pharmacy expenditures if drugs with high inflation-penalized sales percentages were to have similar price increases to those with low inflation-penalized sales percentages and vice versa.

## Methods

This analysis used historical Medicare Part D prescribing and pricing data to estimate the sales percentage of each drug subject to inflation penalties in the 340B program and to examine the association of inflation penalties with price changes. Per the decision guidance of the US Department of Health and Human Services, this study was exempt from informed consent because it did not involve health care records and all data were publicly available. This study followed the Strengthening the Reporting of Observational Studies in Epidemiology (STROBE) reporting guideline for cross-sectional studies.

I performed an analysis of pooled cross-sectional time-series data (ie, panel data) from January 1, 2013, to December 31, 2017, to model the association between differences in inflation-penalized sales percentages and annual drug price changes over the study period. The Medicare Provider Utilization and Payment Data file from 2013 through 2017 was used to identify prescriber-level claims for individual drugs.^12^ This data set is organized by national provider identifiers (NPIs). Each NPI is linked to drug-level claims information for all drugs prescribed by an individual prescriber. Each NPI drug entry includes the number of beneficiaries for whom the drug was prescribed by a specific prescriber, the number of claims, the number of 30-day prescription fills, the total number of days prescribed, and the total drug invoice cost as well as other data. For privacy concerns, the number of beneficiaries who received the prescription was omitted if fewer than 11 beneficiaries received the prescription from a given prescriber.

Summary table data sets from the Medicare Provider Utilization and Payment Data–Part D Prescriber database for 2013 through 2017 were used to match each NPI to the full address information of each organization.^12^ The Office of Pharmacy Affairs Information System of the Health Resources and Services Administration, which includes a database of 340B-covered entities, was used to identify facilities that were actively enrolled in the 340B program from January 1, 2013, to December 31, 2017.^13^ Only facilities that were actively enrolled for the entirety of each study year were considered eligible 340B facilities for that year.

The United States Pharmacopeia (USP) Medicare Model Guidelines, version 8.0,^14^ was used to identify the therapeutic class for brand-name drugs. Pricing data were obtained from the Medicare Part D Drug Spending Dashboard of historical data,^15^ which provides volume-weighted annual unit-price data in a consistent format for Medicare drug use over time. These pricing data reflect pharmacy reimbursement prices and do not include any rebates to pharmacy benefit managers (PBMs).

Data matching was performed using an approach previously detailed elsewhere.^16^ In brief, the use of each drug by NPI was matched to each organization’s address. Those data were matched to 340B program status based on the registered address of the organization. Brand-name drugs were matched to their USP categories by the first word in the brand name. If a drug had multiple USP category designations, 1 category was selected based on a clinical review of the drug’s primary indication, and drugs that were matched to both a Medicare Part D protected class and an additional category were assigned to the protected-class category. Generic drugs were excluded from the analysis because they were not subject to inflation penalties until 2017^17^; vaccines and equipment were also excluded because they are not eligible for discounts in the 340B program.^18^

The analysis considered annual inflation-penalized sales percentages from 2013 through 2017. Price change data considered both the price change from the previous year and the price change into the next year, depending on the model used, and included annual mean price data from 2012 through 2018. Drugs were only included for the years in which they were used in the Medicare program and had no generic competition.

The outcome variable was price change, which comprised either the price change from the previous year or the price change into the next year, depending on the model used. The indicator variable was the inflation-penalized sales percentage, and USP category was included as a potential confounding variable. Price change was centered at 0.

Brand-name drugs that were used by at least 5000 beneficiaries in a given year were included in the study. Sensitivity analyses were performed using different drug inclusion criteria, including analyses of all drugs used (ie, a semibalanced panel that included all years for which a drug was marketed) and drugs used by at least 1000 beneficiaries annually, at least 10 000 beneficiaries annually, and at least 50 000 beneficiaries annually. Analyses were conducted with and without outliers; outliers were calculated based on 3 median average deviations within the outcome variable (price change).

### Statistical Analysis

Ordinary least squares linear panel regression models were used for analysis. Between-effects regression analysis was the principal analytic method, although fixed-effects (within-regression) and random-effects modeling were also performed. The between-effects method was selected because this method considers differences in pricing behavior across drugs relative to inflation-penalized sales percentage rather than the price association of a change in inflation-penalized sales percentage for a given drug. This method is appropriate when the prescribing location data for a given drug remain relatively similar from year to year, reflecting an inflation-penalized sales percentage that is generally consistent across time. By using a between-effects model, which uses the mean inflation-penalized sales percentage of a drug over the period to estimate the mean price change of the drug over the period, I modeled differences in pricing behavior for drugs that generally have lower inflation-penalized sales percentages relative to drugs with generally high inflation-penalized sales percentages. For this model, I used price change from the previous year as the dependent variable because this variable modeled the mean price change within the period for which inflation-penalized sales percentage data were available (ie, the mean inflation-penalized sales percentage from 2013-2017 was used to estimate mean price changes in the 2013-2017 period; using the next-year price change would have modeled mean price changes in the 2014-2018 period). Next-year price change was included in other models and in the sensitivity analysis. The USP category was included as a potential confounding variable, and the results were calculated with and without the use of this variable.

I also performed fixed-effects (within-regression) modeling, which considered the ways in which a change in inflation-penalized sales percentage across time for a given drug was associated with price changes. In this model, I used price change into the next year as the outcome variable, hypothesizing that manufacturers set their price for the next year based on the actual inflation-penalized sales percentage in the current year (ie, inflation-penalized sales in 2013 would be used to estimate the price change in 2014). Because the USP category is constant for a particular drug, this variable could not be included in the fixed-effects model. A random-effects model was also used, which presented a matrix-weighted mean of the coefficients from both the between-effects and fixed-effects models. For both models, 2-sided hypothesis testing was used; results were considered significant at *P* < .05.

To estimate savings associated with inflation penalties and potential savings if a more expansive inflation penalty were instituted, I separated the data into high and low inflation-penalized sales percentage clusters using k-means cluster analysis. I then applied the difference in the mean inflation-penalized sales percentage between the 2 clusters to the between-effects regression model coefficient to estimate the greater price increases for the high inflation–penalty cluster that would occur in the absence of the inflation penalty, and I similarly estimated the lower prices for the low inflation–penalty cluster that would occur with a more expansive inflation penalty. Savings were estimated only for the 2013 to 2017 period and were not adjusted for higher or lower prices before the study period or other potential changes in manufacturer behavior associated with a broader inflation penalty.

Sensitivity analyses were performed using different drug use inclusion criteria and varying the use of previous-year or next-year price changes in the regression methods. Sensitivity analyses were performed with and without outliers. All analyses were performed from January 1 to February 28, 2020, using Stata software, version 16 (StataCorp LLC).

## Results

Among the 2148 brand-name drugs included in the Medicare Part D database, 606 drugs were used by more than 5000 beneficiaries per year (Table 1). For both samples, the estimated sales percentage subject to inflation penalties was 12.1%.After removing outliers, the analysis included 583 drugs, with a mean of 3.3 years of data per drug (of 5 total years in the study period). This analytic subset comprised 85.1% of all drug pharmacy expenditures and 90.4% of drug use over the period.

**Table 1.** Proportion of Medicare Part D Drug Use Subject to Inflation Penalties, 2013 to 2017

Data subset	Drugs, No.	Observation,No. of drugs by y	Inflation-penalized use, d^a^	Total use, d	Inflation-penalized use, %	Total spending, $
All brand drugs	2148	7593	3 536 141 360	29 294 384 040	12.1	485 937 825 494
Drugs used by >5000 beneficiaries	606	2212	3 483 449 760	29 001 117 091	12.0	438 415 763 744
Drugs after removing outliers	583	1929	3 197 376 251	26 478 688 206	12.1	413 409 380 340

^a^ Inflation-penalized use was measured by sales to 340B-covered entities, which receive a discount based on price increases higher than the rate of inflation.

For the analytic subset in which a between-effects model was used, an increase in sales percentage subject to inflation penalties was associated with a lower price increase compared with the previous year (between-effects coefficient, −0.110; 95% CI, −0.169 to −0.052; *P* < .001) (Table 2). This association remained when controlling for USP category (between-effects coefficient, −0.114; 95% CI, −0.205 to −0.023; *P* = .01). Therefore, a drug with a 10% higher mean sales percentage subject to inflation penalties over the period was associated with a 1.1% lower mean annual price increase.

**Table 2.** Linear Panel Regression Analysis for Medicare Part D Drug Price Increases Relative to Sales Percentage Subject to Inflation Penalties, 2013 to 2017

Model	Between -effects coefficient	*P* value	95% CI	Observations^a^	Drug groups
Price change from previous year, between-effects model					
Outliers included	−0.401	.008	−0.697 to −0.105	2212	606
Outliers removed	−0.110	<.001	−0.169 to −0.052	1929	583
Controlled for USP category					
Outliers included	−0.498	.03	−0.956 to −0.040	2212	606
Outliers removed	−0.114	.01	−0.205 to −0.023	1929	583
Price change into next year, fixed-effects model					
Outliers included	−0.895	<.001	−1.281 to −0.508	2249	603
Outliers removed	−0.380	<.001	−0.466 to −0.294	1945	564
Price change from previous year, random-effects model					
Outliers included	−0.399	.002	−0.653 to −0.145	2212	606
Outliers removed	−0.092	<.001	−0.140 to −0.043	1929	583
Controlled for USP category					
Outliers included	−0.492	.005	−0.833 to −0.151	2212	606
Outliers removed	−0.083	.009	−0.146 to −0.021	1929	583

Abbreviation: USP, US Pharmacopeia.

^a^ Variation in sample size in the fixed-effects model occurs by using the next year, rather than previous year, to model the price change.

Using k-means cluster partitioning, drugs in the low inflation–penalized sales percentage cluster had a mean sales percentage subject to inflation penalties of 10.2% and a mean previous-year price increase of 10.3%. Among the drugs in the high inflation–penalized sales percentage cluster, the mean sales percentage subject to the inflation penalty was 30.2% and the mean previous-year price increase was 8.2%. The Figure graphs these data, including the linear estimates from the regression model and the cluster means.

**Figure.**
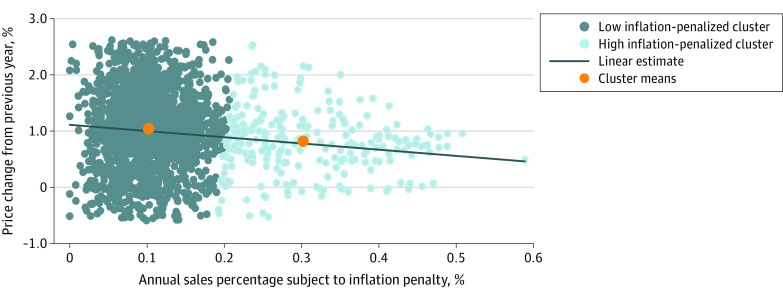
Price Change vs Sales Percentage Subject to Inflation Penalties, 2013 to 2017

In the fixed-effects model, an increase in sales percentage subject to inflation penalties was associated with a lower price increase in the next year (fixed-effects coefficient, −0.380; 95% CI, −0.466 to −0.294; *P* < .001). Therefore, for a given drug, a 10% increase in the sales percentage subject to inflation penalties compared with the previous year was associated with a 3.8% lower price increase in the next year. In the random-effects model, an increase in the sales percentage subject to inflation penalties was associated with a lower price increase compared with the previous year (random-effects coefficient, −0.092; 95% CI, −0.140 to −0.043; *P* < .001); this association remained when controlling for USP category (random-effects coefficient, −0.083; 95% CI, −0.146 to −0.021; *P* = .009).

If an inflation penalty were not instituted and drugs in the high inflation–penalized sales percentage cluster performed similarly to those in the low inflation–penalized sales percentage cluster with regard to price increases, Medicare Part D pharmacy expenditures on the drugs in the analytic subset were estimated to have been $7.1 billion higher, which represented a 1.71% decrease in spending (Table 3). If a broader inflation penalty were instituted and the drugs in the low inflation–penalized sales percentage cluster had price increases consistent with those of the high inflation–penalized sales percentage cluster, Medicare Part D pharmacy expenditures were estimated to have been $17.2 billion lower, which represented a 4.17% spending decrease. These estimates assumed that drug demand was relatively inelastic to price (ie, stable regardless of price) owing to medical necessity and that sales volumes would therefore not change.

**Table 3.** Estimated Medicare Part D Savings From Inflation Penalties, 2013 to 2017

Year	Total spending on included drugs, $, billions^a^	Savings achieved from lower price increases on drugs with high inflation–penalized sales percentages^b^		Savings possible if drugs with low inflation–penalized sales percentages had lower price increases	
		$, Billions	Decrease in spending, %	$, Billions	Decrease in spending, %
2013	60.3	0.1	0.17	1.1	1.84
2014	68.2	0.5	0.76	2.0	3.00
2015	86.4	1.1	1.23	3.4	3.92
2016	95.3	2.1	2.22	4.8	5.04
2017	103.3	3.3	3.18	5.9	5.69
Total	413.4	7.1	1.71	17.2	4.17

^a^ Includes drugs used by more than 5000 Part D beneficiaries in 1 year, after excluding outliers based on price changes. Outliers were excluded based on 3 median average deviations.

^b^ Savings were based on pharmacy expenditures at list price and did not account for rebates.

The results of the sensitivity analyses are displayed in Table 4. The findings were consistent across all sensitivity analyses, and the effect sizes and statistical significance were similar to those of the analytic subset across analyses. The sensitivity analysis using a higher beneficiary-inclusion threshold (n = 50 000) indicated a greater association between the inflation-penalized sales percentage and price change compared with the analytic subset (between-effects coefficient, −0.257; 95% CI, −0.412 to −0.103; *P* = .001), suggesting that the association identified in the analytic subset may have underestimated the implications for drugs used by a large population.

**Table 4.** Sensitivity Analyses of Linear Panel Regression Model of Medicare Part D Drug Price Increases Relative to Sales Percentage Subject to Inflation Penalties, 2013 to 2017

Model	Observations	Drug groups	Between-effects coefficient	95% CI	*P* value
**Entire data set**					
Price change from previous year, between-effects model					
Outliers included	7593	2148	−0.119	−0.221 to −0.018	.021
Outliers removed	6651	2030	−0.041	−0.062 to −0.020	<.001
Controlled for USP category					
Outliers included	7593	2148	−0.082	−0.190 to 0.027	.14
Outliers removed	6651	2030	−0.036	−0.057 to −0.014	.001
Price change into next year, fixed-effects model					
Outliers included	7790	2123	−0.164	−0.254 to −0.074	<.001
Outliers removed	6638	1832	−0.029	−0.046 to −0.013	.001
Price change from previous year, random-effects model					
Outliers included	7593	2148	−0.072	−0.142 to −0.003	.04
Outliers removed	6651	2030	−0.026	−0.040 to −0.011	<.001
Controlled for USP category					
Outliers included	7593	2148	−0.049	−0.121 to 0.023	.18
Outliers removed	6651	2030	−0.022	−0.036 to −0.007	.003
**1000 Beneficiaries**					
Price change from previous year, between-effects model					
Outliers included	3937	1084	−0.276	−0.424 to −0.129	<.001
Outliers removed	3410	1042	−0.094	−0.137 to −0.050	<.001
Controlled for USP category					
Outliers included	3937	1084	−0.303	−0.489 to −0.118	.001
Outliers removed	3410	1042	−0.097	−0.152 to −0.042	.001
Price change into next year, fixed-effects model					
Outliers included	4020	1077	−0.362	−0.563 to −0.160	<.001
Outliers removed	3450	986	−0.149	−0.202 to −0.095	<.001
Price change from previous year, random-effects model					
Outliers included	3937	1084	−0.294	−0.418 to −0.171	<.001
Outliers removed	3410	1042	−0.070	−0.103 to −0.037	<.001
Controlled for USP category					
Outliers included	3937	1084	−0.288	−0.435 to −0.141	<.001
Outliers removed	3410	1042	−0.067	−0.104 to −0.029	<.001
**10 000 Beneficiaries**					
Price change from previous year, between-effects model					
Outliers included	1654	450	−0.507	−1.118 to 0.103	.10
Outliers removed	1451	432	−0.107	−0.173 to −0.041	.001
Controlled for USP category					
Outliers included	1654	450	−0.631	−1.707 to 0.445	.25
Outliers removed	1451	432	−0.112	−0.232 to 0.008	.07
Price change into next year, fixed-effects model					
Outliers included	1678	449	−0.931	−1.481 to −0.380	.001
Outliers removed	1457	424	−0.562	−0.676 to −0.448	<.001
Price change from previous year, random-effects model					
Outliers included	1654	450	−0.620	−1.029 to −0.210	.003
Outliers removed	1451	432	−0.111	−0.168 to −0.055	<.001
Controlled for USP category					
Outliers included	1654	450	−0.714	−1.208 to −0.221	.005
Outliers removed	1451	432	−0.114	−0.195 to −0.032	.006
**50 000 beneficiaries**					
Price change from previous year, between-effects model					
Outliers included	807	230	−0.430	−0.760 to −0.101	.01
Outliers removed	712	225	−0.257	−0.412 to −0.103	.001
Controlled for USP category					
Outliers included	807	230	−1.031	−1.453 to −0.609	<.001
Outliers removed	712	225	−0.257	−0.478 to −0.036	.02
Price change into next year, fixed-effects model					
Outliers included	813	232	−1.330	−1.874 to −0.786	<.001
Outliers removed	711	220	−0.817	−0.988 to −0.647	<.001
Price change from previous year, random-effects model					
Outliers included	807	230	−0.500	−0.769 to −0.232	<.001
Outliers removed	712	225	−0.264	−0.377 to −0.151	<.001
Controlled for USP category					
Outliers included	807	230	−0.691	−1.019 to −0.363	<.001
Outliers removed	712	225	−0.264	−0.399 to −0.129	<.001

Abbreviation: USP, US Pharmacopeia.

## Discussion

The association between a greater sales percentage subject to inflation penalties and a lower price increase is consistent with the general theory of inflation penalties, which posits that, when presented with a financial penalty for price increases that are higher than the rate of inflation, drug manufacturers would prefer to implement lower price increases to avoid incurring the penalty. Notably, this observation is inconsistent with the proposition that manufacturers subject to inflation penalties are likely to implement greater price increases than they would in the absence of penalties; across all models, the data suggest no association between higher inflation penalties and greater price increases. This finding provides empirical support for the theory that increases in mandatory discounts are not associated with cost-shifting to all purchasers through higher list prices, as the ability to shift costs would suggest that manufacturers were underpricing their products relative to what the market could sustain, which is inconsistent with a profit-maximizing strategy.^19,20^

Manufacturers increase drug prices for myriad reasons, including to increase revenues, establish reference prices for new drugs,^21^ and appeal to insurers and PBMs.^22^ If a drug manufacturer implements a drug price increase but rebates the value of the increase to an insurer or PBM, this approach can help to reduce insurance premiums by shifting a greater portion of the net costs into beneficiary out-of-pocket spending.^23^ Although the drug manufacturer’s net revenues may be constant in this situation, the benefit to the insurer or PBM can encourage better formulary treatment of the manufacturer’s product, increasing the manufacturer’s net revenues.

Therefore, an inflation penalty will only discourage price increases if it sufficiently counteracts the broader incentives to increase prices. In the absence of any PBM rebates, an inflation penalty would not be expected to be associated with a price reduction, as the manufacturer may simply pay the rebate and achieve the same net price while gaining the value of a higher reference price for future products. However, PBMs generally require that price increases higher than a certain threshold are rebated through contractual arrangements known as price-protection clauses.^24^ Therefore, an inflation penalty will be most successful in discouraging price increases when a manufacturer is required to pay both the inflation penalty and the PBM price protection rebate, ensuring that price increases are actually associated with decreases in the manufacturer’s net revenues.

For example, assume a $90 drug increases to $100, and $9 of that price increase is higher than the rate of inflation. If the manufacturer pays a $9 rebate to either Medicare or the PBM, it will retain the same net revenue as it would by limiting its price increase to $91. But if the manufacturer must pay a $9 rebate to both Medicare (by law) and the PBM (by contract), it would have lower net revenue than that obtained from a limited price increase to $91. A similar association has been observed in pricing patterns for hepatitis C treatments, in which high PBM rebates coupled with substantial discounts in the 340B program made it more profitable for manufacturers to decrease their list prices rather than to offer equivalent discounts via a rebate.^16^

The data in the present analysis indicated that, although drugs with substantial sales percentages subject to inflation penalties had lower price increases compared with other drugs, their price increases remained higher than the rate of inflation. This finding suggests that the inflation penalty present in the 340B program may not sufficiently counteract other manufacturer incentives to increase prices. As policy makers analyze approaches to new inflation penalties, it will be important to consider whether such penalties have consequences for enough of the market to discourage price increases or whether another policy, such as a multiplier,^25^ may be necessary.

Although this study only considered Medicare Part D pharmacy reimbursement prices, which do not include PBM rebates, these prices are important for Medicare beneficiaries. Medicare beneficiaries are generally required to pay 25% of drug pharmacy prices in cost-sharing payments,^26^ meaning that they do not benefit at the pharmacy counter from any price-protection rebates provided to PBMs (although these rebates do reduce premiums for beneficiaries). This type of rebate practice shifts drug costs from premiums to beneficiary cost-sharing, increasing the proportion of costs incurred by beneficiaries with substantial use of those drugs.^23^ Therefore, policies that restrain increases in list prices are suitable for reducing point-of-sale costs for beneficiaries; such reductions in cost may be associated with increases in drug adherence and broader savings for the Medicare program.^27^

### Limitations

This study has several limitations. First, errors could have occurred in the data-matching process, and not all prescriptions by physicians in a 340B-eligible organization may be filled in the 340B program. Although these errors could have produced incorrect estimates of the inflation-penalized sales percentage for a given drug, they are unlikely to be systematically biased and therefore would not have consequences for the overall association between price change and inflation-penalized sales percentage.

Because this study used Medicare prescribing data only, the findings may not reflect the total sales percentage subject to inflation penalties for a given drug if non-Medicare drug use were to substantially differ based on the prescribing site (and therefore 340B eligibility). However, unless this difference in prescribing site were nonsystematic, it would be unlikely to have implications for the general association found. These issues may limit the generalizability of results beyond the population of individuals with Medicare Part D.

This study did not consider new brand-name drug competition as a separate confounding variable in the model. However, by controlling for USP category, the model accounted for the general level of therapeutic competition within a class. Furthermore, other research has indicated that the introduction of new brand-name drug competition is not associated with a decrease in the list prices of existing brand-name drugs,^28^ suggesting it is unlikely that the introduction of new competitors was associated with the results.

## Conclusions

In this cross-sectional study, brand-name drugs with higher sales percentages subject to inflation penalties were associated with lower annual price increases. No data were found to indicate that inflation penalties or discounts in the 340B program were associated with higher price increases, suggesting that mandatory inflation-based price concessions are not associated with higher list-price increases. Even when encountering substantial inflation penalties, drug manufacturers remain likely to implement price increases that are higher than the rate of inflation. Policy makers may need to consider the extent of inflation penalties necessary to counteract this behavior when designing policies.
