# Errors in Figure

**DOI:** 10.1001/jamanetworkopen.2020.26259

**Published:** 2020-10-16

In the Original Investigation titled “Association Between the Percentage of US Drug Sales Subject to Inflation Penalties and the Extent of Drug Price Increases,”^1^ published September 11, 2020, there were errors in the Figure. The axis tick marks given as proportions should have been given as percentage values. This article was corrected.^1^
